# Aryloxy Ionic Liquid-Catalyzed Homogenous Esterification of Cellulose with Low-Reactive Acyl Donors

**DOI:** 10.3390/polym15020419

**Published:** 2023-01-13

**Authors:** Akina Yoshizawa, Chie Maruyama, Samuel Budi Wardhana Kusuma, Naoki Wada, Kosuke Kuroda, Daisuke Hirose, Kenji Takahashi

**Affiliations:** 1 Graduate School of Natural Science and Technology, Kanazawa University, Kakuma-machi, Kanazawa 920-1192, Japan; 2 Department of Chemistry, Faculty of Mathematics and Natural Science, Universitas Negeri Semarang, Semarang City 50229, Indonesia

**Keywords:** ionic liquid, aryloxy anion, pyridinolate, biobased polymer, cellulose, esterification

## Abstract

Ionic liquids (ILs) are recyclable, non-volatile, and can dissolve cellulose, a natural polymer that is insoluble in versatile solvents. Therefore, ILs have been used to modify cellulose. However, 1-ethyl-3-methylimidazolium acetate (EmimOAc), a commercially available IL often used to dissolve and modify cellulose to prepare cellulose-based materials, causes the undesired introduction of an acetyl group derived from the acetate anion of EmimOAc onto the hydroxy group of cellulose during esterification. In this study, for cellulose esterification, we prepared aryloxy ILs as non-carboxylate-type and basic ILs, which can theoretically prevent the undesired introduction of an acyl group from the IL onto the hydroxy group of cellulose. The optimized 1-ethyl-3-methylimidazolium 2-pyridinolate (Emim2OPy) and mixed solvent system achieved rapid cellulose esterification (within 30 min) with an excellent degree of substitution (DS) value (up to >2.9) derived from the employed low-reactive vinyl esters and bio-based unsaturated aldehydes, without any undesired substituent introduction from side reactions.

## 1. Introduction

Currently, the growing awareness about the environmental impacts of petroleum-based materials is necessitating the development of their replacements, such as bio-based polymers, to reduce reliance on petroleum resources in the polymer industry [1]. Cellulose is a polysaccharide with a linear chain of *β*-1,4 linked D-glucose units and is the most abundant renewable bio-based polymer in the world [2,3]. Cellulose can be chemically modified into a range of polymeric derivatives with remarkable properties that are suitable for various applications [4]. However, cellulose is insoluble in common organic solvents and water because of the inter- and intramolecular hydrogen bonds between the hydroxyl groups in the glucose unit [3]. Therefore, many cellulose modifications, such as esterification [5], etherification [6,7], and silylation [8,9], are commonly achieved through heterogeneous routes, and the heterogeneous reaction process in cellulose modification requires overstoichiometric amounts of reactants and acids or bases for activating the hydroxyl groups in cellulose. Furthermore, controlling the degree of substitution (DS) of the resulting modified cellulose derivatives using a heterogonous modification process is difficult. Therefore, cellulose modification under homogeneous reaction conditions is an attractive research area in cellulose chemistry. In 2002, Rogers et al. first demonstrated that molten organic salts with relatively low melting points (<100 °C), called ionic liquids (ILs) [10,11], can dissolve cellulose under mild reaction conditions [12].

**Figure 1:**
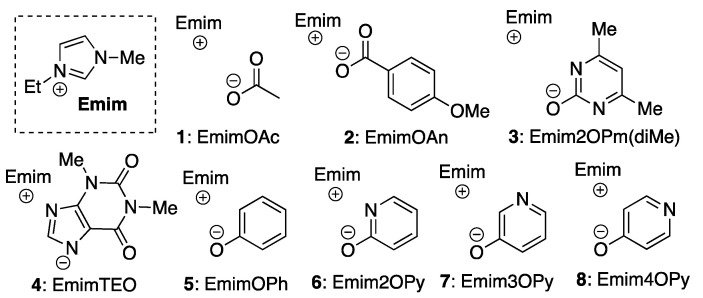
Chemical structures of ILs.

Since their report, chemical modifications of cellulose, such as esterification under homogeneous reaction conditions utilizing ILs, have been reported. The first-generation esterification of cellulose in the presence of ILs is usually performed with highly reactive and corrosive carboxylic acid chlorides or anhydrides [13,14,15,16]. The utilization of acyl chlorides or anhydrides for cellulose esterification leads to high conversion rates but generates environmentally unfriendly halides or useless carboxylic acids as side products. A promising alternative to acyl chloride or anhydrides for the esterification of cellulose is the use of vinyl esters (VEs). In 2015, our group reported the acetylation of cellulose with low-reactive isopropenyl acetate (IPA) in the presence of 1-ethyl-3-methylimidazolium acetate (EmimOAc **1**) as a catalyst and solvent (Figure 1) [17].

**Scheme 1:**
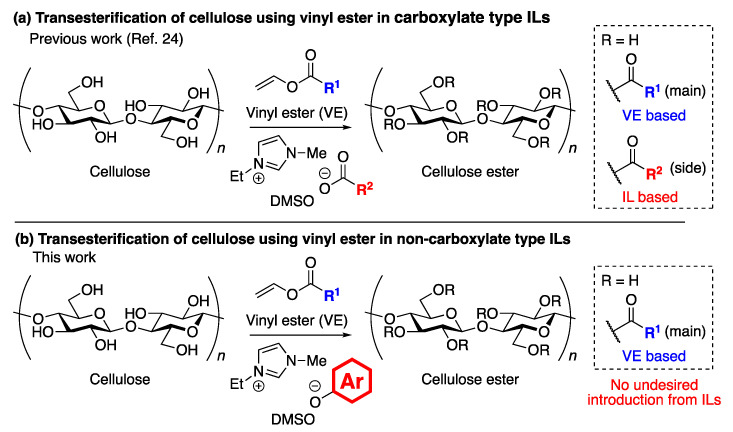
Schematic illustration of the transesterification of cellulose using vinyl esters in ILs. (**a,b**) Transesterification of cellulose using vinyl ester in carboxylate type ILs (**a**) and non-carboxylate type aryloxy ILs (**b**).

This cellulose esterification is a green and efficient reaction that does not require any additional catalyst, such as a strong acid or base, and an environmentally unfriendly acyl donor. However, for vinyl esters, except IPA, this process causes the undesired introduction of an acetyl group derived from the acetate anion of EmimOAc **1** (Scheme 1a) [18]. The introduction of undesired substituents onto cellulose might alter the thermal [19] and chemical [20,21] properties of the resulting cellulose derivatives. Therefore, undesired anion contamination as ester substituents from ILs must be avoided during cellulose esterification.

Previous studies have predicted that the mixed acid anhydride is formed from the IL anion and acyl donor. Then, the hydroxy groups of cellulose nucleophilically attack the carbonyl carbon of the mixed acid anhydride, leading to side reactions [22,23]. Based on this finding, the design of ILs with different carboxylate anions was investigated in our previous reports to prevent the generation of mixed acid anhydrides and proceed with the desired acylation with high selectivity [24,25]. ILs with optimized anion substituents and bulkiness, for example, 1-ethyl-3-methylimidazolium *p*-anisate (EmimOAn **2**), were able to suppress the formation of mixed acid anhydrides while maintaining high cellulose solvation and transesterification reactivity [24]. However, in the transesterification reaction of cellulose using carboxylate-type ILs, it is theoretically undeniable that mixed acid anhydrides are formed from the anions of ILs and VE, and it is difficult to completely prevent the introduction of undesired acyl groups from the anions of the ILs.

Therefore, in this study, we investigated a non-carboxylate-type basic IL that mainly includes an aryloxy anion that cannot form mixed acid anhydrides in the esterification reaction between cellulose and VEs (Scheme 1b). The cellulose solvation and catalytic activity of the prepared ILs were evaluated for trans- and oxidative esterification with low-reactivity acyl donors.

## 2. Materials and Methods

### 2.1. Materials

1-Ethyl-3-methylimidazolium chloride (EmimCl), DMSO anhydrous, and cellulose (Avicel^®^PH-101, particle size < 50 μm) were obtained from Sigma-Aldrich Co. LLC. (St. Louis, MO, USA). EmimCl was used after recrystallization twice from acetonitrile. The number-average degree of polymerization of Avicel was calculated to be 105 [22]. EmimOAc **1** was obtained from Nippon Nyukazai Co., Ltd. (Tokyo, Japan). 4-Methoxybenzoic acid, 2-hydroxypyridine, 3-hydroxypyridine, 4-hydroxypyridine, theophylline, 4,6-dimethyl-2-hydroxypyrimidine, vinyl pivalate, vinyl propionate, vinyl laurate, and cinnamaldehyde were purchased from Tokyo Chemical Industry (TCI, Tokyo, Japan). Reichardt’s Dye, *N,N*-diethyl-4-nitroaniline and 4-nitroaniline were purchased from Sigma-Aldrich Co. LLC. (St. Louis, MO, USA). Other chemicals were obtained from commercial sources and were used without further purification.

### 2.2. Instruments

^1^H and ^13^C NMR spectra were recorded at 600 MHz on a JNM-ECA 600 spectrometer (JEOL Ltd., Tokyo, Japan) in deuterated solvents. Chemical shifts (δ, ppm) are reported in ppm relative to tetramethylsilane (δ 0.00) for the ^1^H NMR and to chloroform (δ 77.0) for the ^13^C NMR measurements. Fourier transform-infrared (FT-IR) spectra were recorded using a Thermo Fisher Scientific Nicolet IS10 (Thermo Fisher Scientific Inc., Waltham, MA, USA) spectrometer equipped with an attenuated total reflection (ATR) unit at room temperature. Mass spectra were recorded in fast atom bombardment (FAB) mode using a JMS-700 (JEOL Ltd., Tokyo, Japan). The UV–vis spectrum was measured at room temperature on a UV-Cary 300 Spectrometer (Agilent, Santa Clara, CA, USA).

### 2.3. Preparation of ILs from EmimCl [24,26,27]

EmimOAn **2** [24], 1-ethyl-3-methylimidazolium phenoxide (EmimOPh **5**) [26] and 1-ethyl-3-methylimidazolium 2-pyridinolate (Emim2OPy **6**) [26] were prepared using the reported method from our group. 1-Ethyl-3-methylimidazolium 3-pyridinolate (Emim3OPy **7**), 1-ethyl-3-methylimidazolium 4-pyridinolate (Emim4OPy **8**), 1-Ethyl-3-methylimidazolium theophylinate (EmimTEO **4**) and 1-ethyl-3-methylimidazolium 4,6-dimethyl-2-hydroxypyrimidinolate (Emim2OPm(diMe) **3**) were synthesized according to conventional protocols, as shown below [26].

Potassium hydroxide (85%, 4.94 g, 68.2 mmol)/2-propanol (120 mL) and equal amount of EmimCl (10 g, 68.2 mmol) as 2-propanol (20 mL) solutions were prepared. Both solutions were cooled to −60 °C. The cooled solutions were mixed and filtered twice to remove precipitated potassium chloride. The filtrate contained 1-ethyl-3-methylimidazolium hydroxide (EmimOH) dissolved in 2-propanol. EmimOH in the solution was neutralized with an equal amount of corresponding hydroxy aryls or theophylline in 2-propanol (10 mL) solution. The reaction mixture was filtered using a glass fiber filter (Advantec, GA-55, 21 mm), 2-propanol was evaporated, and the filtrate was dried in vacuo at room temperature. The resultant ILs were quantitatively obtained as yellow, brown, sticky oils, or white solids.

### 2.4. Solubility of Cellulose in ILs

The solubility of cellulose in a mixed solution of ILs and DMSO (1/20, molar ratio) was tested using the following method: The cellulose was pre-dried for four hours in vacuo at 60 °C. At 80 °C, the pre-dried cellulose (15 mg) was added to a solution (1 mL) containing DMSO and the respective IL, and kept for 15 min. After 15 min, the dissolution of cellulose in the reaction solution was visually confirmed. If complete dissolution of the cellulose was confirmed, an additional 15 mg of cellulose was added. The final process was continued until the saturation of cellulose in the DMSO/IL mixture.

### 2.5. General Procedure for the Transesterification of Cellulose 

As a typical case, cellulose (240 mg, [AGU (anhydrous glucose unit)] = 1.48 mmol, [OH] = 4.44 mmol) was mixed with the Emim2OPy **6** (470 mg, 2.29 mmol) and dried for three hours in vacuo at 80 °C. After complete drying, anhydride DMSO (5.2 mL) was added, and the mixture was stirred for one hour at 80 °C under an argon atmosphere. Vinyl pivalate (10.5 mL, 71.6 mmol) was added, and the reaction mixture was stirred for 24 h at 80 °C. The resulting solution was poured into a large amount of 50% methanol solution, and precipitated cellulose ester was collected by filtration. The polymer was purified by precipitation (from acetone solution into water) to obtain cellulose pivalate as a white powder (DS = 2.99, 283 mg, 48%).

### 2.6. General Procedure for the Oxidative Esterification of Cellulose

The synthesis of cellulose esters via oxidative esterification was conducted using a previously reported method [26,28]. As a typical example, cellulose (120 mg, [AGU] = 0.740 mmol, and [OH] = 2.22 mmol) was mixed with the Emim2OPy **6** (455 mg, 2.22 mmol) and dried for three hours in vacuo at 80 °C. After complete drying, anhydride DMSO (3.15 mL) was added, and the mixture was stirred for one hour at 60 °C under an argon atmosphere. In the next step, after adding cinnamaldehyde (0.6 mL, 2.22 mmol), the reaction mixture was stirred at 60 °C for 24 h. After the reaction, the resulting solution was poured into methanol. The polymer was purified by reprecipitation (from acetone solution into methanol) to obtain cellulose phenylpropionate as a pale yellowish powder (DS = 2.78, 312 mg, 79%).

### 2.7. Evaluation of the DS in Cellulose Esters

The DS in the cellulose esters was determined by ^1^H NMR spectroscopy conducted in DMSO-*d_6_*, acetone-*d_6_*, or CDCl_3_ depending on the solubility of the product in deuterated solvents and the overlap between the target and solvent peaks. To determine the DS values of the low-modified cellulose derivatives, the DS values were calculated after perbenzoylation or peracetylation. The DS values determined from the desired substituents (DS_main_) of the modified cellulose derivatives were calculated using the following Equation (1):

DS_main_ = (I_main_/X)/(I_AGU_/7) (1)

The DS_main_ values for cellulose pivalate, propionate, laurate, and phenylpropionate were calculated from the integrals of the corresponding peaks (I_main_) at approximately 1.3–0.9 ppm (X = 9), 1.3–1.0 ppm (X = 3), 1.0−0.7 ppm (X = 3) and 7.8−6.5 ppm (X = 5), respectively, and the AGU peaks (I_AGU_) at approximately 5.5−3.0 ppm. The DS values determined from the undesired substituents (DS_side_) were calculated using Equation (2):

DS_side_ = (I_side_/X)/(I_AGU_/7) (2)

DS_side_ values of cellulose esters derived from EmimOAc **1** and EmimOAn **2** were calculated from the integrals of the peaks (I_side_) at approximately 2.2−1.7 ppm (X = 3) and 8.3–6.8 ppm (X = 4), corresponding to a methyl group and an aromatic ring, respectively, and the areas of AGU signals (I_AGU_) at 3.0–5.5 ppm.

### 2.8. General Procedure for Per-Benzoylation of the Synthesized Cellulose Ester

The DS_side_ values for cellulose esters synthesized by transesterification were determined after perbenzoylation to produce accurate DS_side_ values. The synthesized cellulose ester (50 mg, [AGU] = 0.308 mmol, calculated as cellulose) was dissolved in *N,N*-dimethylacetamide (DMAc, 3 mL). Benzoic acid (1.85 mmol, 226 mg), 4-dimethylaminopyridine (DMAP, 1.85 mmol, 226 mg), and 1-(3-dimethylaminopropyl)-3-ethylcarbodiimide hydrochloride (EDC HCl, 1.85 mmol, 355 mg) were added, and the reaction mixture was stirred at room temperature for 24 h. After the reaction, the perbenzoylated cellulose ester was precipitated from methanol (30 mL), collected, and washed with methanol three times. The collected product was dried overnight in vacuo in the oven at 70 °C.

### 2.9. General Procedure for Per-Acetylation of the Synthesized Cellulose Esters

The synthesized cellulose ester (50 mg, [AGU] = 0.308 mmol, calculated as cellulose) was dissolved in DMAc (3 mL). DMAP (1.85 mmol, 226 mg), acetic acid (1.85 mmol, 0.106 mL), and EDC HCl (1.85 mmol, 355 mg) were added, and the reaction mixture was stirred at room temperature for 24 h. After the reaction, the peracetylated cellulose ester was precipitated from methanol and collected. The collected product was dried overnight in vacuo in the oven at 70 °C.

### 2.10. Kamlet–Taft Parameters

From the spectroscopic data, the acidity (*α*) and basicity (*β*) were calculated using equations previously published by Kamlet et al. [29,30].

## 3. Results and Discussion

### 3.1. ILs Synthesis and Cellulose Dissolution in ILs

Non-carboxylate-type ILs that do not form mixed acid anhydride intermediates were designed to synthesize ILs with high cellulose solvation and without the undesired introduction of anions into the hydroxy groups on cellulose (Figure 1). ILs containing various non-carboxylate anions were prepared to investigate the influence of IL on transesterification of cellulose with VE. Next, the solubility of cellulose in the prepared ILs was tested. A mixed-solvent system of DMSO and IL has been reported to enhance the performance of ILs in the dissolving cellulose [31,32] and accelerate some cellulose-modification reactions [22]. Thus, the solubility of cellulose in mixed-solvent systems containing non-carboxylate-type ILs and DMSO was tested and compared with those reported for mixed-solvent systems containing carboxylate-type ILs and DMSO [24]. ILs **2–8**, with melting points below 100 °C, were synthesized via the anion-exchange method using potassium hydroxide [24,27]. ILs **3** and **5–8**, with aryloxide ions, were prepared from EmimCl and its corresponding aromatic hydroxyl compounds. EmimTEO **4** was synthesized from EmimCl and theophylline, a bio-based alkaloid. IL **4** does not contain an aryloxide structure, but it exhibits basicity similar to that exhibited by other aryloxy ILs. Therefore, the cellulose solubility and catalytic ability of **4** were also investigated, as with the other non-carboxylate-type ILs in this study.

The solubility of cellulose, in the range of 15–75 mg in a mixed-solvent system of synthesized ILs and DMSO (1/20 molar ratio), was examined at 80 °C (Table 1) [31]. In our previous report [24], the solubility of cellulose in a mixed-solvent system of IL/DMSO was determined to depend on the basicity of the anion of the IL. Bulky anions effectively prevented undesired anion introduction onto cellulose, but the steric hindrance of the IL anions decreased its efficiency in dissolving cellulose.

Based on the pKa value of the conjugated acid [33], which is an indicator of anion basicity (Table 1), the basicity of the anions in the synthesized ILs was in the order **6** (12.0) > **3** (10.3) > **7** (9.15) > **4** (8.60) > **8** (5.19) > **1** (4.79) > **2** (4.47). The pKa values of the conjugated acid of anions are shown in parentheses. The trend in the solubility of cellulose in the carboxylate ILs was **1** > **2**, similar to the order of the basicity of the anions of the ILs (Table 1, entries 1 and 2). Although ILs **3** and **4** had sufficient basicity for cellulose dissolution (Table 1, entries 3 and 4), these two ILs showed low cellulose solubilities, probably due to their large anion size. To improve cellulose solvation, EmimOPh **5** (the pKa value of the conjugated acid is 9.86), which has a smaller size, higher basicity anion, and is a better acceptor of hydrogen bonds, was synthesized. However, the synthesized **5** was not sufficiently stable for cellulose modification [26,34,35]. To stabilize the negative charge on the anions of ILs, we tested EmimOPys **6–8** which have a nitrogen atom at the 2-4 position of the aromatic ring. These **6-8** showed sufficient cellulose solvation and stability for cellulose modification. In the case of imidazolium-type ILs with 2OPy-4OPy anions, the cellulose solvation in the mixed solvent system of ILs and DMSO was also in the order **6** (12.0) > **7** (9.15) > **8** (5.19), corresponding to the order of basicity of the anion.

To further discuss the solubility of cellulose in the synthesized ILs, the polarity of the ILs was evaluated through the Kamlet-Taft (KT) polarity evaluation method based on the solvatochromism of dyes, using parameters such as hydrogen bond basicity and acidity (*β* and *α* value, respectively) [36]. The KT parameters of Emim2OPy **6**, which has the highest cellulose solvation, and Emim4OPy **8**, which has the lowest cellulose solvation among EmimOPys **6–8**, were calculated from the solvatochromic behavior of three dyes: 4-nitroaniline, *N,N*-diethyl-4-nitroaniline, and Reichardt’s Dye 30 (Table 2) [29,37]. To discuss the KT parameters considering the cellulose solvation conditions, this measurement was performed in DMSO. Due to the breaking of hydrogen bonds between cellulose molecules, imidazolium-type ILs with *β* > 0.8 show the ability to dissolve cellulose [38]. Mixed-solvent systems containing DMSO with Emim2OPy **6** and Emim4OPy **8** (IL/DMSO = 1/20 (molar ratio)) showed relatively high *β* values of 1.04 and 0.93, respectively, similar to that of pure EmimOAc **1** (0.95–1.09) [38,39]. This observation supports the high solubility of cellulose in both ILs. However, the difference between the *β* values was slightly smaller than that between the solubility values, indicating that the KT parameters may not be the main factors responsible for solubility. It was difficult to obtain reproducible KT parameters for Emim2OPy **6** and Emim4OPy **8** under neat conditions without dilution with DMSO, probably due to their high viscosities.

### 3.2. Catalytic Activity of Synthesized ILs in Transesterification of Cellulose

**Figure 2:**
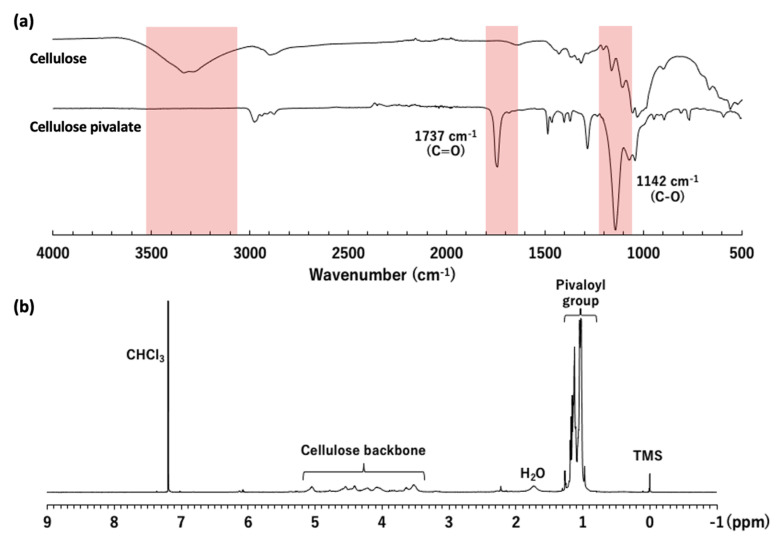
(**a**) ATR-mode FT-IR spectra of the original cellulose (Avicel^®^PH-101) and the cellulose pivalate prepared in the presence of Emim2OPy **6** (Table 1, entry 5). (**b**) ^1^H NMR spectra of the resulting cellulose pivalate in CDCl_3_ measured at room temperature.

**Figure 3:**
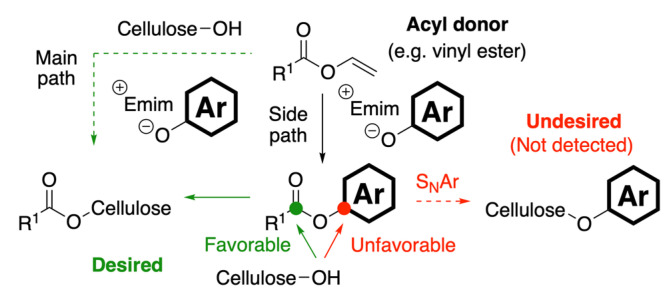
Predicted mechanism of the side pathways in transesterification of cellulose in the presence of aryloxy ILs.

The reactivity of the prepared ILs in the transesterification reaction was confirmed using vinyl pivalate (VPiv, **9**), an acyl donor that is difficult to introduce and causes easy introduction of undesired acyl groups derived from the ILs anions (Table 1). At first, the transesterification of cellulose was carried out using a mixed solvent system of DMSO and Emim2OPy **6**, which had the highest solubility for cellulose among the prepared ILs at 80 °C (Table 1, entry 5). The reaction solution was homogeneous throughout the process, indicating that the ILs effectively solvated cellulose in this system. After the reaction, the resulting cellulose derivative was recovered from the reaction mixture by precipitation in the 50% methanol solution (*v/v*). The structure of the resulting cellulose derivative was confirmed using infrared (IR) and ^1^H NMR spectroscopy. The IR absorption spectrum of the obtained cellulose derivative showed an absorption band at 1737 cm^−1^ derived from the C=O stretching vibrations of the carbonyl group (Figure 2a), supporting the progress of cellulose esterification. The ^1^H NMR spectrum revealed proton peaks derived from a pivaloyl group at 0.9–1.3 ppm and a cellulose backbone at 3.0–5.5 ppm (Figure 2b), the resulting cellulose derivative was confirmed to be cellulose pivalate. Interestingly, there were no proton peaks at 6.5–8.0 ppm derived from an aromatic ring in the ^1^H NMR spectrum, indicating that the undesired substituents derived from 2OPy anion were not introduced into the hydroxyl group of the cellulose. Even an aryl ester formed by the nucleophilic attack of the aryloxy anion of Emim2OPy **6** on VPiv **9** may introduce an aromatic ring onto the hydroxy group of cellulose by aromatic nucleophilic substitution reactions (S_N_Ar). However, this transesterification of cellulose with VPiv **9** in the presence of Emim2OPy **6** proceeded with only the desired transesterification (Figure 3) [22,41].

To determine accurate DS values, the residual hydroxyl groups of the resulting cellulose pivalate were perbenzoylated and peracetylated to determine the DS value of the desired pivaloyl group (DS_main_) and undesired group (DS_side_), followed by ^1^H NMR measurements. In the ^1^H NMR spectrum of the perbenzoylated sample of the obtained cellulose pivalate, a comparison of the integrated values of the proton peaks of the pivaloyl group and the cellulose backbone showed that the DS_main_ value of the cellulose pivalate synthesized using Emim2OPy **6** reached DS_main_ = 2.99. This result demonstrated that pure cellulose pivalate with an almost complete substitution (DS_main_ > 2.9) was successfully synthesized with a mild acyl donor.

Subsequently, we tested the transesterification of cellulose with VPiv **9** in the presence of other ILs **1–8** (except **5**) prepared in this study (Table 1). In all experiments, DS_main_ and DS_side_ values were obtained using the same method as transesterification with Emim2OPy **6**. However, the DS_side_ value of the transesterification in EmimOAc **1** was calculated from ^1^H NMR spectra measured after per-benzoylation. From all the experiments, it was clear that the transesterification proceeded smoothly in all the mixed solvent systems of DMSO and the prepared imidazolium-type ILs **1–8** (except **5**), yielding cellulose pivalates with high DS_main_ values of 2.43 to 2.99. As previously reported, the transesterification reaction of cellulose with VPiv **9** in carboxylate-type ILs **1** and **2** (Table 1, entries 1 and 2) resulted in anion contamination from ILs with ester substituents (DS_side_ = 0.54 and 0.03) [24]. The introduction of undesired anions from ILs **1** and **2** was confirmed by the ^1^H NMR of the obtained cellulose pivalate. Furthermore, EmimOAc **1** limited the DS_main_ value of cellulose pivalate, and it never even reached 2.5 due to the side reaction derived from its acetate anion. On the other hand, the transesterification reactions in non-carboxylate-type ILs **3–8** (except **5**) showed much higher DS_main_ values of 2.73–2.99 than those in EmimOAc **1** (Table 1, entries 3–7). Notably, the results show that the selectivity of transesterification in non-carboxylate type ILs was almost 100%, indicating that these ILs prevented the undesired introduction of the aryl oxide group from anions of ILs.

### 3.3. Optimization of Reaction Conditions and Kinetics of Cellulose Esterification

**Figure 4:**
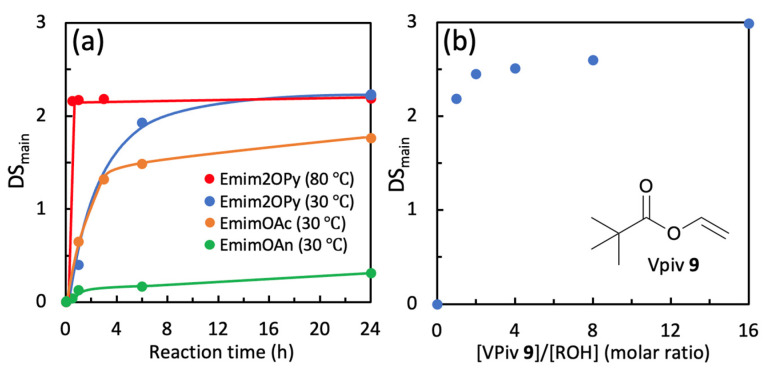
(**a**) Time-dependent change in DS_main_ value in the synthesis of cellulose pivalate. Reaction conditions: [OH]/[IL]/[DMSO]/[VPiv **9**] = 1/0.5/17/1 (molar ratio), Ar atmosphere. (**b**) Effect of the amount of VPiv **9** on cellulose OH groups. Reaction conditions: [OH]/[Emim2OPy **6**]/[DMSO] = 1/0.5/17 (molar ratio), 24 h, 80 °C, Ar atmosphere.

Next, the kinetics of transesterification using VPiv **9** was investigated. Figure 4a shows the time-dependent change in DS_main_ values of cellulose pivalate prepared using one equivalent of VPiv **9** per hydroxy group of cellulose in a mixed solvent system of DMSO with EmimOAc **1**, EmimOAn **2**, and Emim2OPy **6** at 30 and 80 °C. The transesterification of cellulose and VPiv **9** in a mixed solvent of DMSO and EmimOAn **2** at 30 °C resulted in a DS_main_ of only 0.17 for the cellulose pivalate obtained after 6 h (Figure 4a, green). Even after extending the reaction time to 24 h, DS_main_ was 0.31 and little progress was made, meaning that the catalytic activity of EmimOAn **2** was very low at room temperature due to its steric hindrance. EmimOAc **1**, which has a smaller carboxylate, exhibited relatively higher catalytic activity at 30 °C than EmimOAn **2**. Cellulose pivalate obtained from the reaction mixture in the mixed solvent system of DMSO and EmimOAc **1** after 24 h showed a better DS_main_ value (1.76) (Figure 4a, orange). 

In contrast, transesterification in the presence of Emim2OPy **6** for 24 h afforded the desired cellulose ester with a higher DS_main_ value (2.23) than the reactions in EmimOAc **1** and EmimOAn **2** without any anion introduction (Figure 4a, blue). This result indicates that the catalytic activity of Emim2OPy **6** in transesterification is high, even at a low temperature of 30 °C. Furthermore, when cellulose was esterified at 80 °C in a mixed solvent system of Emim2OPy **6** and DMSO, it required less than 30 min to reach a saturated DS_main_ value (2.16), suggesting satisfactory fast kinetics of cellulose esterification (Figure 4a, red). These results show that Emim2OPy **6** has higher catalytic activity in the transesterification of cellulose with VPiv **9** than that of previously reported carboxylate ILs.

Next, the amount of VPiv **9** was varied to maximize the DS_main_ value of the main ester group in the obtained cellulose ester (Figure 4b). Although VPiv **9** is a bulky acyl donor, this transesterification in the presence of Emim2OPy **6** using one equivalent of VPiv **9** per hydroxyl group of cellulose provided the synthesized cellulose pivalate with a high DS_main_ value (2.19). The synthesized cellulose pivalate (DS_main_ = 2.19) exhibited sufficient solubility in various lipophilic solvents and molding processability. The DS_main_ values of the obtained cellulose pivalate were improved from 2.19 to 2.60 by increasing the amount of VPiv **9** from one equivalent to eight equivalents per hydroxy group of cellulose, and a fully substituted cellulose pivalate (DS_main_ = >2.9) was obtained by using 16 equivalents of vinyl pivalate.

### 3.4. Scope of Vinyl Esters

Emim2OPy **6** which had the highest cellulose solvation in the above study (Table 1) and the optimized reaction conditions were applied to the transesterification of cellulose with different vinyl esters such as vinyl propionate (VPro **10**) and vinyl laurate (VL **11**) (Table 3). After perbenzoylation of the synthesized cellulose esters, the DS_main_ values of the cellulose esters were determined by ^1^H NMR spectroscopy using the same method as in Table 1. In these three experiments, the DS_main_ values of the cellulose esters showed almost full substitution on the side chain (pivalate: DS_main_ = 2.96 (entry 1), propionate: DS_main_ = 2.85 (entry 3), and laurate: DS_main_ = 2.96 (entry 4)) without any anion contamination of Emim2OPy **6** as ether substituents based on ^1^H NMR spectra (Figures S9 and S10). Even in transesterification with bulky vinyl pivalate, the introduction of the desired ester group with complete selectivity was successful, although a side reaction was observed when EmimOAc **1** was used (DS_side_ = 0.54) (Table 3, entry 2). 

The solubilities of the synthesized cellulose esters using VPiv, VPro, and VL were tested in various solvents, and these three cellulose esters with high DS_main_ values showed high solubility in various organic solvents such as chloroform, tetrahydrofuran, and acetone (Table S1). The high solubilities of the synthesized cellulose derivatives in various organic solvents indicated easy processing of the obtained cellulose derivatives [42,43].

### 3.5. Effect of the IL Species on Oxidative Esterification of Cellulose

Recently, we reported a synthetic method for fully bio-based cellulose esters using bio-based unsaturated aldehydes such as cinnamon-based cinnamaldehyde **12** as acyl donors based on different mechanisms of transesterification [26,28]. This reaction proceeds via an oxidative esterification mechanism in which the carbon–carbon double bond (C=C) works as an intramolecular oxidant and is an environmentally benign method because there is no leaving group, unlike conventional esterification methods, including transesterification, that use acid chloride, anhydride, and condensation reagents. Under mild reaction conditions, oxidative esterification hardly proceeds without *N*-heterocyclic carbene (NHC)-based catalytic ability of the ionic liquid [26,28,44]. Therefore, aldehydes are regarded as inactive acyl donors in conventional cellulose esterification without an ionic liquid. However, the effect of the aryloxy IL structure on this new green cellulose esterification method is still not clear. Synthesized aryloxy ILs EmimOPys **6–8** showing high cellulose solvation were applied to oxidative cellulose esterification for discussion of cellulose solvation and reactivity.

**Figure 5:**
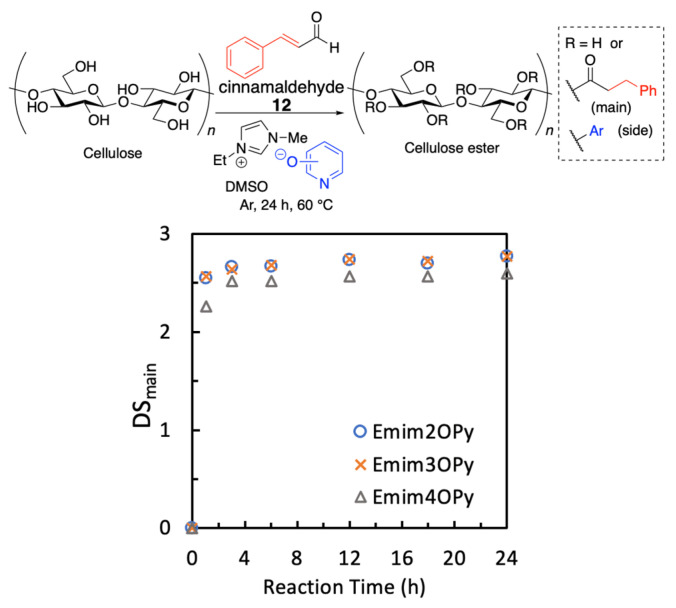
Time-dependent change in DS_main_ value in the synthesis of cellulose phenylpropionate. Reaction conditions: [OH]/[cinnamaldehyde **12**]/[IL]/[DMSO] = 1/1/1/20 (molar ratio), 24 h, 60 °C, Ar atmosphere.

To investigate the kinetics of the oxidative esterification of cellulose with cinnamaldehyde **12**, time-dependent DS_main_ value changes in the resulting cellulose phenylpropionate were evaluated [26,28]. After the dissolution of cellulose in the mixed solvent of synthesized EmimOPys **6–8** and DMSO, cinnamaldehyde **12** was added to the reaction mixture and stirred at 60 °C (Figure 5). After the reaction, the resulting cellulose esters were recovered and washed with methanol. The DS_main_ values were determined from ^1^H NMR spectra of the synthesized cellulose phenylpropionate after per-acetylation. The DS_main_ value of cellulose phenylpropionate was calculated from the integrated peak ratio between the cellulose backbone and aromatic ring in ^1^H NMR spectra. In the oxidative esterification of cellulose in EmimOPys **6–8**, the undesired introduction of ether groups from the anions of the ionic liquid due to side reactions was not observed (Figures S11–S13). In the case of the oxidative esterification of cellulose in the mixed solvent of Emim2OPy **6** and DMSO, the DS_main_ value of the synthesized cellulose phenylpropionate was quite high (2.55) during the first hour. Subsequently, extending the reaction time to 24 h only increased the DS_main_ value of cellulose phenylpropionate to a maximum of 2.78, and extending the reaction time did not significantly contribute to improving the DS_main_ value. The oxidative esterification of cellulose in the presence of Emim3OPy **7** showed almost the same behavior as that in the presence of Emim2OPy **6**. On the other hand, in the oxidative esterification of cellulose using Emim4OPy, the DS_main_ value was lower than that when using Emim2OPy **6** or Emim3OPy **7**. Emim4OPy **8** catalyzed oxidative esterification of cellulose also afforded the desired cellulose phenylpropionate after one hour, but its DS_main_ value was relatively small (2.27) and the reaction rate was slower than that of Emim2OPy **6** or Emim3OPy **7**. These results indicate that aryloxy ILs with higher cellulose solvation (Emim2OPy **6** or Emim3OPy **7**) are excellent catalysts and solvents for cellulose esterification with low-reactive acyl donors, including oxidative esterification and transesterification.

## 4. Conclusions

For the precise synthesis of cellulose esters using mild low-reactive acyl donors in ILs, we prepared non-carboxylate-type ILs that theoretically negate the formation of mixed acid anhydrides that cause the undesired introduction of anions as acyl groups onto hydroxy groups. Furthermore, we evaluated the effects of the basicity and bulkiness of the anions of ILs on cellulose solvation and catalytic activity for cellulose esterification. Aryloxy ILs, which have a relatively small anion with high basicity, show high cellulose solvation and effectively catalyze cellulose esterification. In a mixed solvent system of DMSO and aryloxy-type ILs, especially 1-ethyl-3-methylimidazolium 2-pyridinolate (Emim2OPy), cellulose esters could be produced with a high DS_main_ value of up to >2.9 without any undesired introduction of an acyl group onto the hydroxy group of cellulose by transesterification of cellulose with low-reactive vinyl esters. Furthermore, a series of ILs also promoted the oxidative esterification of cellulose and afforded the target product with a high DS_main_ value (up to 2.78) without introducing an IL anion-based structure due to side reactions. The results of this study show that the synthesis of cellulose esters with high substitutions and structural uniformity in ILs can be achieved by two different esterification reactions using aryloxy-type ILs that can completely suppress the formation of side reactions.

## Figures and Tables

**Table 1:**
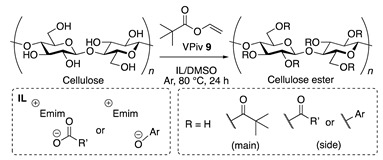
Effect of ILs in transesterification of cellulose *^a^*.

Entry	IL	*pK_a_^b^*	Solubility *^c^*	DS_main_ *^d^*	DS_side_ *^d^*	Selectivity (%)
1	**1**	4.79	<75	2.43	0.54	82
2	**2**	4.47	<45	2.78	0.03	99
3	**3**	10.3	<15	2.85	n.d.	>99
4	**4**	8.60	<15	2.73	n.d.	>99
5	**6**	12.0	<75	2.99	n.d.	>99
6	**7**	9.15	<60	2.85	n.d.	>99
7	**8**	5.19	<30	2.74	n.d.	>99

*^a^* Reaction conditions: [OH]/[IL]/[DMSO]/[VPiv **9**] = 1/0.5/17/16 (molar ratio); for 24 h, 80 °C under Ar atmosphere. *^b^* The pKa value of the conjugated acid. *^c^* Solubility test (mg mL^−1^) in IL/DMSO = 1/20 (molar ratio) solvent system at 80 °C. *^d^* Determined by ^1^H NMR spectroscopy. n.d.: not detected (estimated DS_side_ << 0.03).

**Table 2:** Kamlet–Taft parameters *α* and *β* of solvent.

Entry	Solvents	*α*	*β*
1	Emim2OPy/DMSO (1/20)	0.20	1.04
2	Emim4OPy/DMSO (1/20)	0.26	0.93
3	EmimOAc	0.40	0.95–1.09 [38,39]
4	DMSO	−0.11	0.72 [40]

**Table 3:**
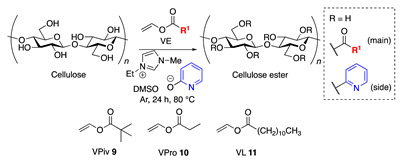
Scope of vinyl ester in the transesterification in the Emim2OPy/DMSO mixed solvent system *^a^*.

Entry	VE	DS_main_ *^b^*	DS_side_ *^b^*	Selectivity (%)
1	**9**	2.96	n.d.	>99
2	**9**	2.43 ^*c*^	0.54 ^*c*^	82 ^*c*^
3	**10**	2.85	n.d.	>99
4	**11**	2.96	n.d.	>99

*^a^* Reaction conditions: [OH]/[Emim2OPy **6**]/[DMSO]/[VE] = 1/0.5/17/16 (molar ratio); for 24 h, 80 °C under Ar atmosphere. *^b^* Determined by ^1^H NMR spectroscopy. *^c^* The esterification was performed in the presence of EmimOAc instead of Emim2OPy.

## Data Availability

Not applicable.

## Supplementary Materials

The following supporting information can be downloaded at: https://www.mdpi.com/article/10.3390/polym15020419/s1, Figure S1: ^1^H NMR spectrum of 1-ethyl-3-methylimidazolium 4,6-dimethyl-2-hydroxypyridinate (Emim2OPm(diMe)) (**3**) in DMSO-*d_6_* at rt.; Figure S2: ^13^C NMR spectrum of 1-ethyl-3-methylimidazolium 4,6-dimethyl-2-hydroxypyrimidinolate (Emim2OPm(diMe)) (**3**) in DMSO-*d_6_* at rt.; Figure S3: ^1^H NMR spectrum of 1-ethyl-3-methylimidazolium theophylinate (EmimTEO) (**4**) in DMSO-*d_6_* at rt.; Figure S4: ^13^C NMR spectrum of 1-ethyl-3-methylimidazolium theophylinate (EmimTEO) (**4**) in DMSO-*d_6_* at rt.; Figure S5: ^1^H NMR spectrum of 1-ethyl-3-methylimidazolium 3-pyridinolate (Emim3OPy) (**7**) in DMSO-*d_6_* at rt.; Figure S6: ^13^C NMR spectrum of 1-ethyl-3-methylimidazolium 3-pyridinolate (Emim3OPy) (**7**) in DMSO-*d_6_* at rt.; Figure S7: ^1^H NMR spectrum of 1-ethyl-3-methylimidazolium 4-pyridinolate (Emim4OPy) (**8**) in DMSO-*d_6_* at rt.; Figure S8: ^13^C NMR spectrum of 1-ethyl-3-methylimidazolium 4-pyridinolate (Emim4OPy) (**8**) in DMSO-*d_6_* at rt.; Figure S9: ^1^H NMR spectrum of cellulose propionate (DS = 2.85) in CDCl_3_ at rt (Table 3, entry 3).; Figure S10: ^1^H NMR spectrum of cellulose laurate (DS = 2.96) in CDCl_3_ at rt (Table 3, entry 4).; Figure S11: ^1^H NMR spectrum of cellulose phenylpropionate (DS = 2.78) synthesized from Emim2OPy **6** in CDCl_3_ at rt (Figure 5, blue, 24 h).; Figure S12: ^1^H NMR spectrum of cellulose phenylpropionate (DS = 2.78) synthesized from Emim3OPy **7** in CDCl_3_ at rt (Figure 5, orange, 24 h).; Figure S13: ^1^H NMR spectrum of cellulose phenylpropionate (DS = 2.60) synthesized from Emim4OPy **8** in CDCl_3_ at rt (Figure 5, gray, 24 h).
