# Supplementary material for: Aryloxy Ionic Liquid-Catalyzed Homogenous Esterification of Cellulose with Low-Reactive Acyl Donors
Source: Polymers (Basel). 2023 Jan 13;15(2):419. doi: 10.3390/polym15020419 (PMC9860810; doi:10.3390/polym15020419)
Supplement: Supplementary file 1 [file polymers-15-00419-s001.zip › polymers-2141408-supplementary.pdf]

(Supporting Information)

Aryloxy ionic liquids-catalyzed homogenous esterification of cellulose with low-reactive acyl donors

Akina Yoshizawa,[a] Chie Maruyama,[a] Samuel Budi Wardhana Kusuma,[a,b] Naoki Wada,[a] Kosuke Kuroda,[a] Daisuke Hirose,[a,]* and Kenji Takahashi[a,]*

[a]*Graduate School of Natural Science and Technology, Kanazawa University, Kakuma-machi, Kanazawa 920-1192, Japan.*

[b]*Department of Chemistry, Faculty of mathematics and Natural Sciences, Universitas Negeri Semarang, Semarang City, Central Java 50229, Indonesia.*

*To whom correspondence should be addressed. E-mail: dhirose@se.kanazawa-u.ac.jp, ktkenji@staff.kanazawa-u.ac.jp.

Preparation of ILs from EmimCl

Potassium hydroxide (85%, 4.94 g, 68.2 mmol)/2-propanol (120 mL) and EmimCl (10 g, 68.2 mmol)/2-propanol (20 mL) solutions were prepared. Both potassium hydroxide and EmimCl solution were cooled to −60°C. The cooled potassium hydroxide and EmimCl solution were mixed and followed by filtration twice to remove the precipitated potassium chloride. 1-Ethyl-3-methylimidazolium hydroxide (EmimOH) was obtained as a 2-propanol solution, and the EmimOH solution was neutralized by an equal amount of corresponding hydroxy aryls or theophyllin in 2-propanol (10 mL) solution. The reaction mixture was filtrated by a glass fiber filter (Advantec, GA-55, 21 mm), evaporation of 2-propanol, and drying in vacuo at room temperature. EmimOPys were quantitatively obtained as a yellow, brown sticky oil or white solid.

1-Ethyl-3-methylimidazolium 4,6-dimethyl-2-hydroxypyrimidinolate (Emim2OPm(diMe)) (3)

Prepared from potassium hydroxide (85%, 4.94 g, 68.2 mmol), EmimCl (10.0 g, 68.2 mmol), 4,6-dimethyl-2-hydroxypyrimidine (8.40 g, 68.2 mmol) and 2-propanol (150 mL). Yellow oil. ^{1}H NMR (600 MHz, DMSO-d_6) δ; 9.95 (s, 1H), 7.84 (s, 1H), 7.75 (s, 1H), 5.78 (s, 1H), 4.24 (q, J = 7.2 Hz, 2H), 3.89 (s, 3H), 1.98 (s, 6H), 1.41 (t, J = 7.2 Hz, 3H). ^{13}C NMR (150 MHz, DMSO-d_6) δ; 166.85, 166.49, 137.93, 124.00, 122.41, 102.79, 44.52, 36.08, 23.60, 15.76. IR (ATR, cm^{-1}) 2964, 1556, 1378, 1334. HRMS FAB (m/z) calcd. for $C_6H_{11}N_2$ ([M]+) 111.0917, found: 111.0924 (cation), and $C_6H_7N_2O$ ([M]−) 123.0564, found: 123.0555 (anion).

1-Ethyl-3-methylimidazolium theophylinate (EmimTEO) (4)

Prepared from potassium hydroxide (85%, 4.94 g, 68.2 mmol), EmimCl (10.0 g, 68.2 mmol), theophylline (12.3g, 68.2 mmol) and 2-propanol (150 mL). Yellow oil. ^{1}H NMR (600 MHz, DMSO-d_6) δ; 9.26 (s, 1H), 7.75 (s, 1H), 7.66 (s, 1H), 7.11 (s, 1H), 4.15 (q, J = 7.2 Hz, 2H), 3.81 (s, 3H), 3.35 (s, 3H), 3.15 (s, 3H), 1.36 (t, J = 7.2 Hz, 3H). ^{13}C NMR (150 MHz, DMSO-d_6) δ; 158.19, 152.33, 150.23, 147.12, 136.94, 124.04, 122.42, 115.59, 44.63, 36.15, 30.41, 27.89, 15.60. IR (ATR, cm^{-1}) 3076, 1689, 1637, 1473. HRMS FAB (m/z) calcd. for $C_6H_{11}N_2$ ([M]$^+$) 111.0917, found: 111.0926 (cation), and $C_7H_7N_4O_2$ ([M]$^-$) 179.0574, found: 179.0570 (anion).

1-Ethyl-3-methylimidazolium 3-pyridinolate (Emim3OPy) (7)

Prepared from potassium hydroxide (85%, 4.94 g, 68.2 mmol), EmimCl (10.0 g, 68.2 mmol), 3-hydroxypyridine (6.49g, 68.2 mmol) and 2-propanol (150 mL). Brown oil. ^{1}H NMR (600 MHz, DMSO-d_6) δ; 9.48 (s, 1H), 7.79 (d, J = 2.4 Hz, 1H), 7.70 (d, J = 1.8 Hz, 1H), 7.41 (d, J = 2.4 Hz, 1H), 7.06 (d, J = 3.6 Hz, 1H), 6.62 (dd, J = 8.4, 4.2 Hz, 1H), 6.24 (d, J = 7.8 Hz, 1H), 4.13 (q, J = 7.2 Hz, 2H), 3.80 (s, 3H), 1.35 (t, J = 7.2 Hz, 3H). ^{13}C NMR (150 MHz, DMSO-d_6) δ; 166.87, 143.02, 137.41, 129.41, 124.08, 124.05, 123.14, 122.50, 44.61, 36.14, 15.66. IR (ATR, cm^{-1}) 2991, 1556, 1470.

HRMS (FAB) calcd for $C_6H_{11}N_2$ ($[M]^+$) 111.0917, found: 111.0919 (cation), and C_5H_4NO ($[M]^-$) 94.0298, found: 94.0292 (anion).

1-Ethyl-3-methylimidazolium 4-pyridinolate (Emim4OPy) (8)

Prepared from potassium hydroxide (85%, 4.94 g, 68.2 mmol), EmimCl (10.0 g, 68.2 mmol), 4-hydroxypyridine (6.49g, 68.2 mmol) and 2-propanol (150 mL). Off-white solid (hygroscopic). ^{1}H NMR (600 MHz, DMSO-d_6) δ; 9.27 (s, 1H), 7.80 (s, 1H), 7.71 (s, 1H), 7.52 (d, J = 6.6 Hz, 2H), 5.84 (d, J = 6.0 Hz, 2H), 4.18 (q, J = 7.2 Hz, 2H), 3.84 (s, 3H), 1.41 (t, J = 7.2 Hz, 3H). ^{13}C NMR (150 MHz, DMSO-d_6) δ; 175.49, 149.26, 137.12 124.10, 122.53, 116.70, 44.62, 36.18, 15.66. IR (ATR, cm^{-1}) 2973, 1583, 1489. HRMS (FAB) calcd for $C_6H_{11}N_2$ ($[M]^+$) 111.0917, found: 111.0921 (cation), and C_5H_4NO ($[M]^-$) 94.0298, found: 94.0295 (anion).

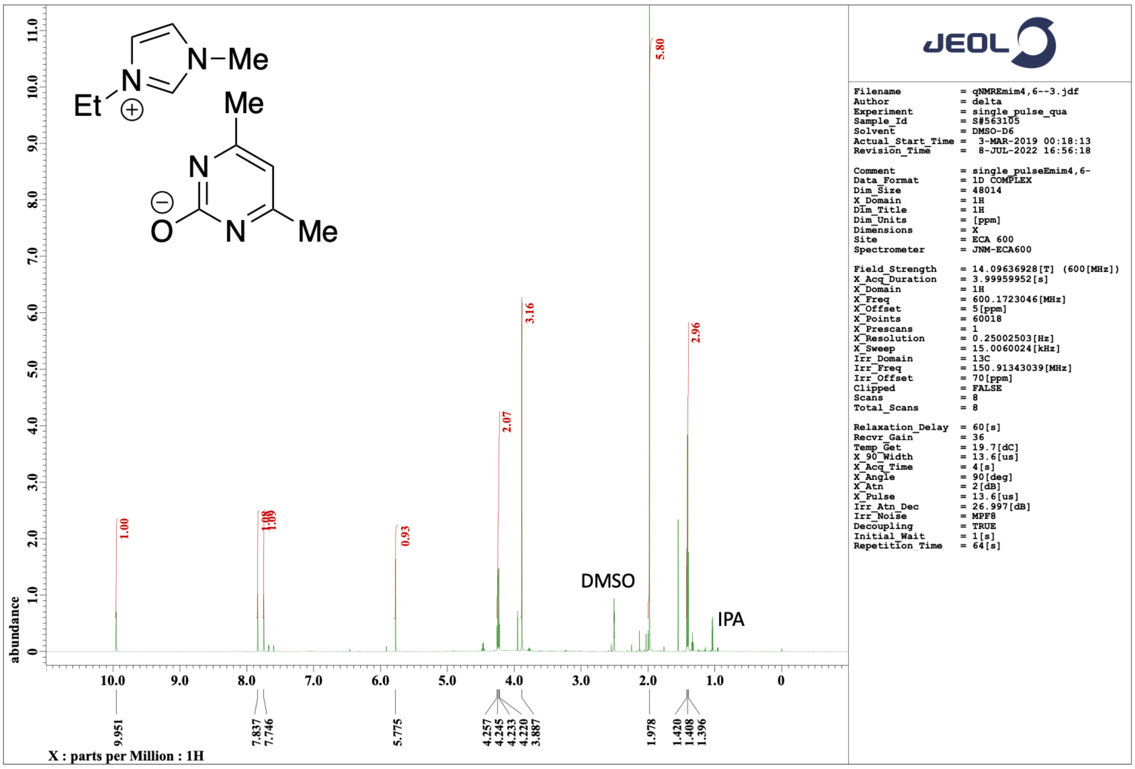

Figure S1. ^{1}H NMR spectrum of 1-ethyl-3-methylimidazolium 4,6-dimethyl-2-hydroxypyrimidinolate (Emim2OPm(diMe)) (**3**) in DMSO-d_6 at rt.

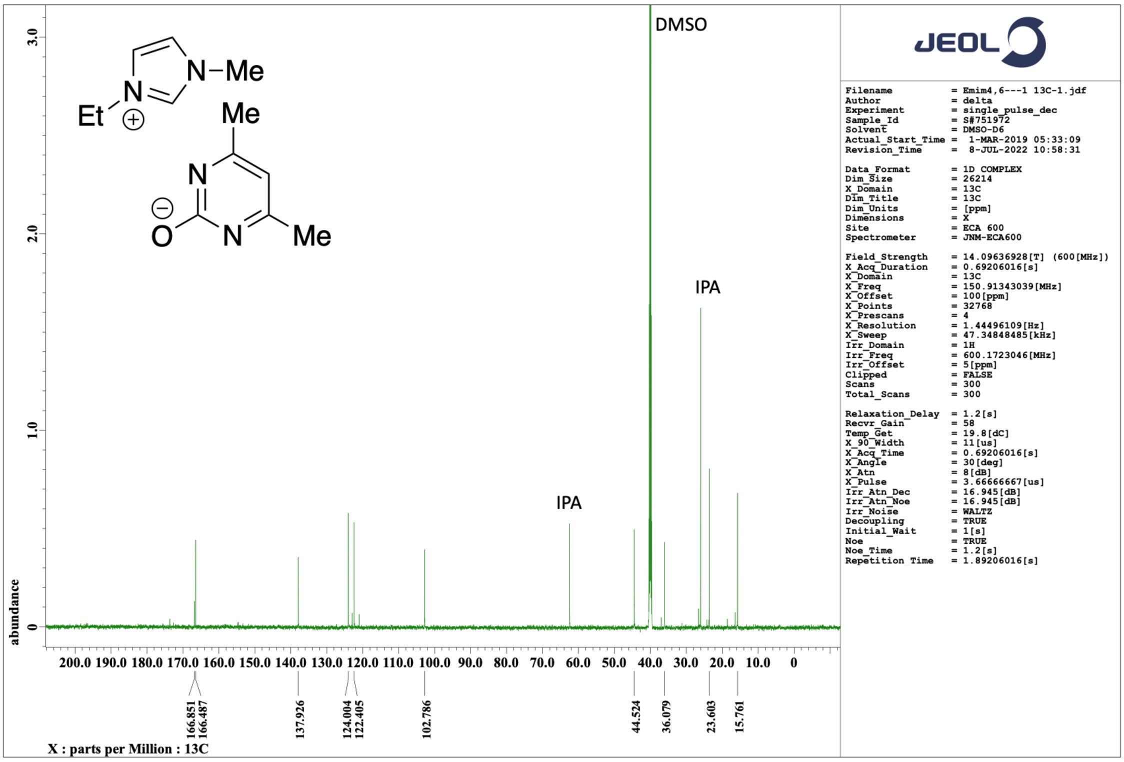

Figure S2. ^{13}C NMR spectrum of 1-ethyl-3-methylimidazolium 4,6-dimethyl-2-hydroxy pyrimidinolate (Emim2OPm(diMe)) (**3**) in DMSO-d_6 at rt.

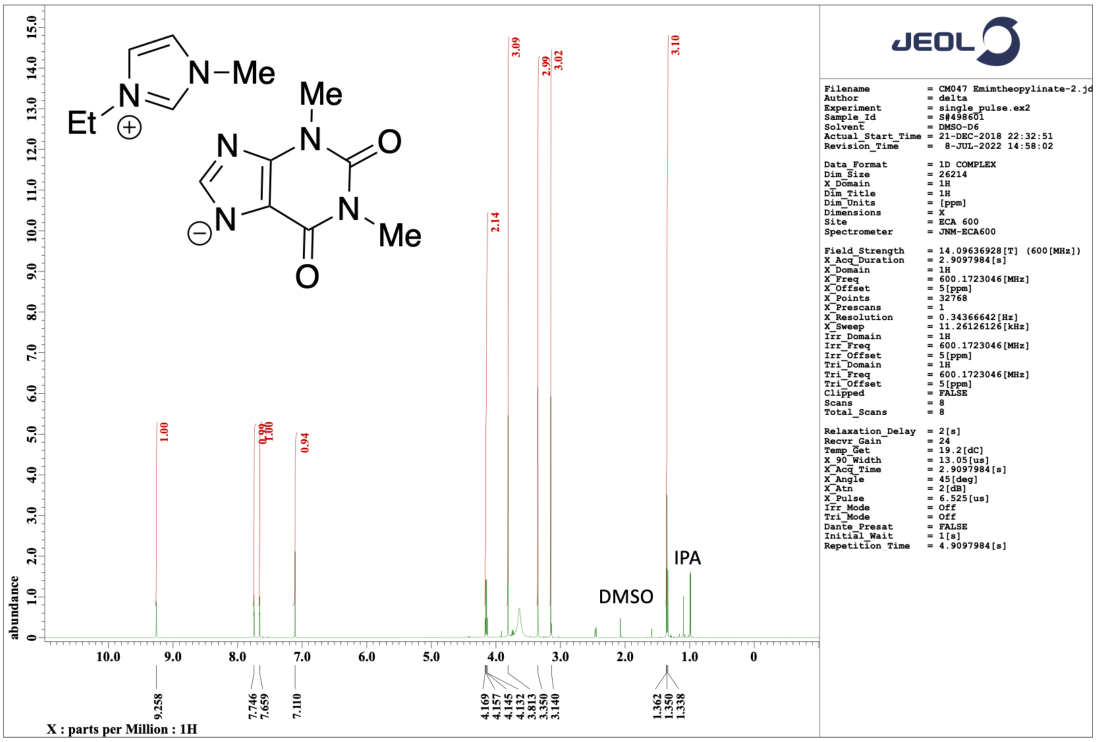

Figure S3. ^{1}H NMR spectrum of 1-ethyl-3-methylimidazolium theophylinate (EmimTEO) (**4**) in DMSO-d_6 at rt.

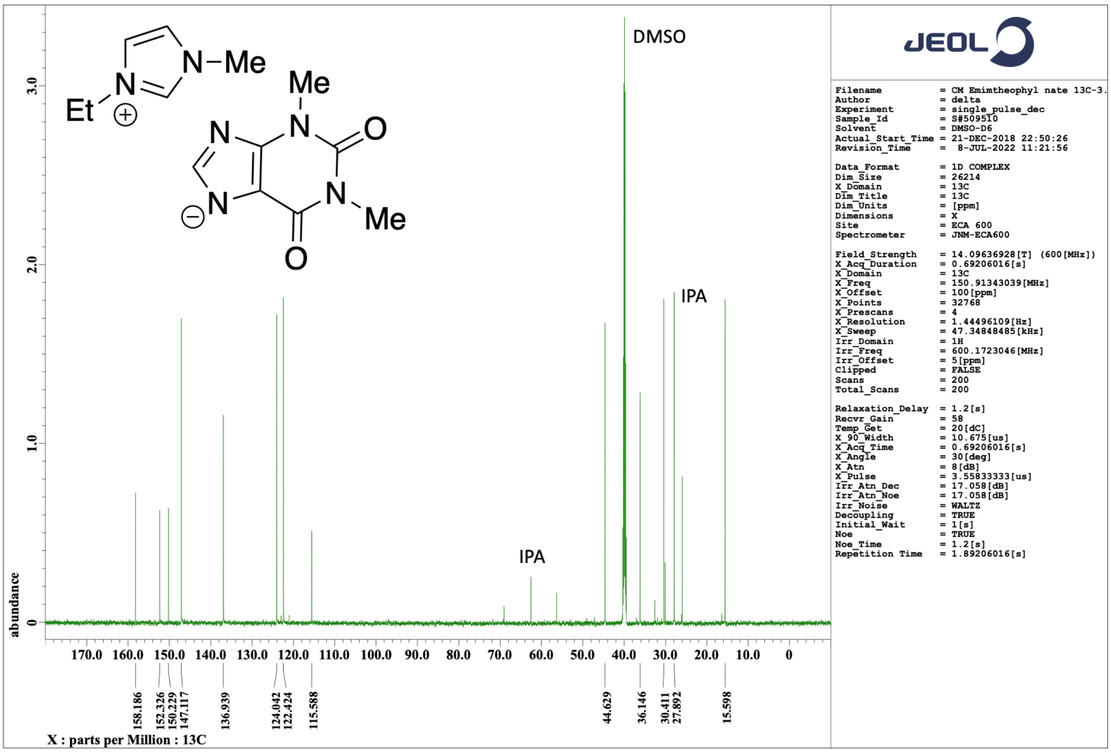

Figure S4. ^{13}C NMR spectrum of 1-ethyl-3-methylimidazolium theophylinate (EmimTEO) (**4**) in DMSO-d_6 at rt.

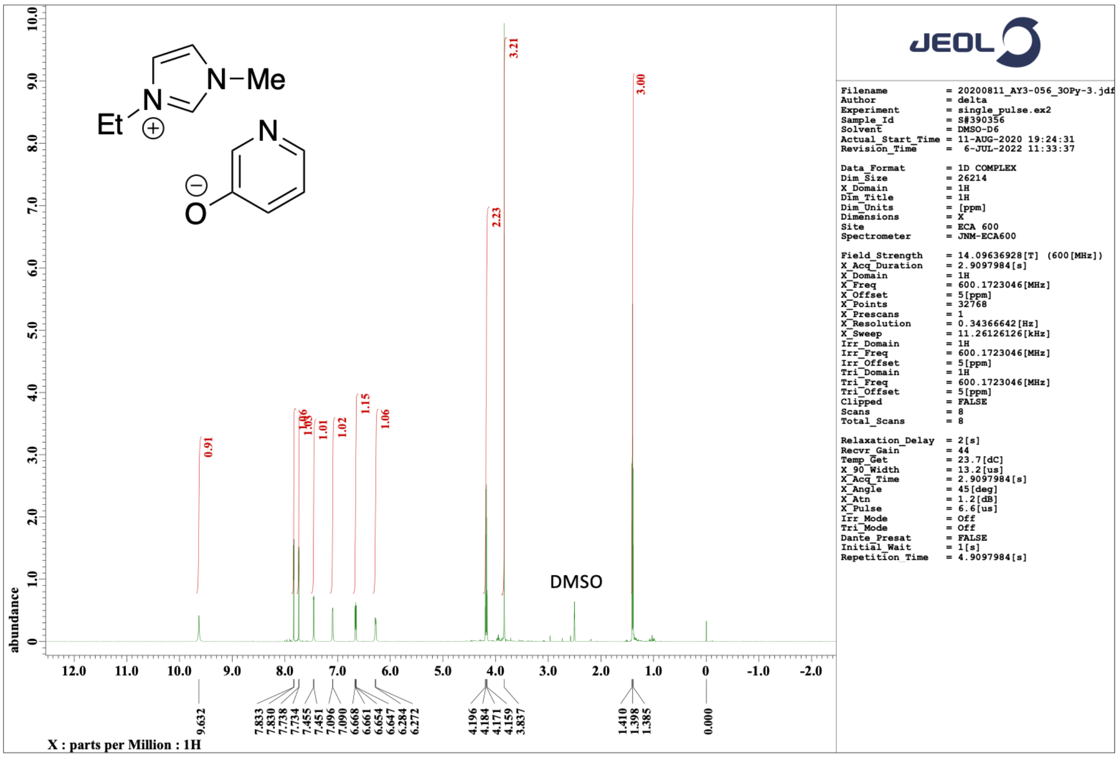

Figure S5. ^{1}H NMR spectrum of 1-ethyl-3-methylimidazolium 3-pyridinolate (Emim3OPy) (**7**) in DMSO-d_6 at rt.

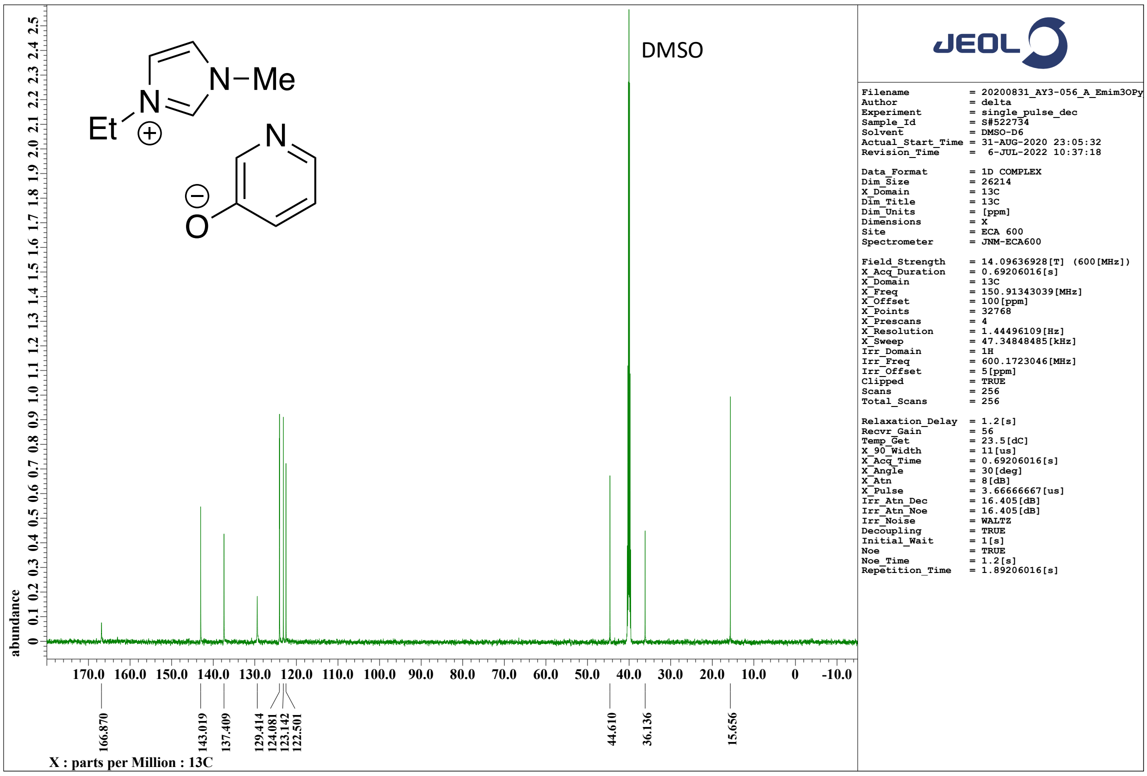

Figure S6. ^{13}C NMR spectrum of 1-ethyl-3-methylimidazolium 3-pyridinolate (Emim3OPy) (**7**) in DMSO-d_6 at rt.

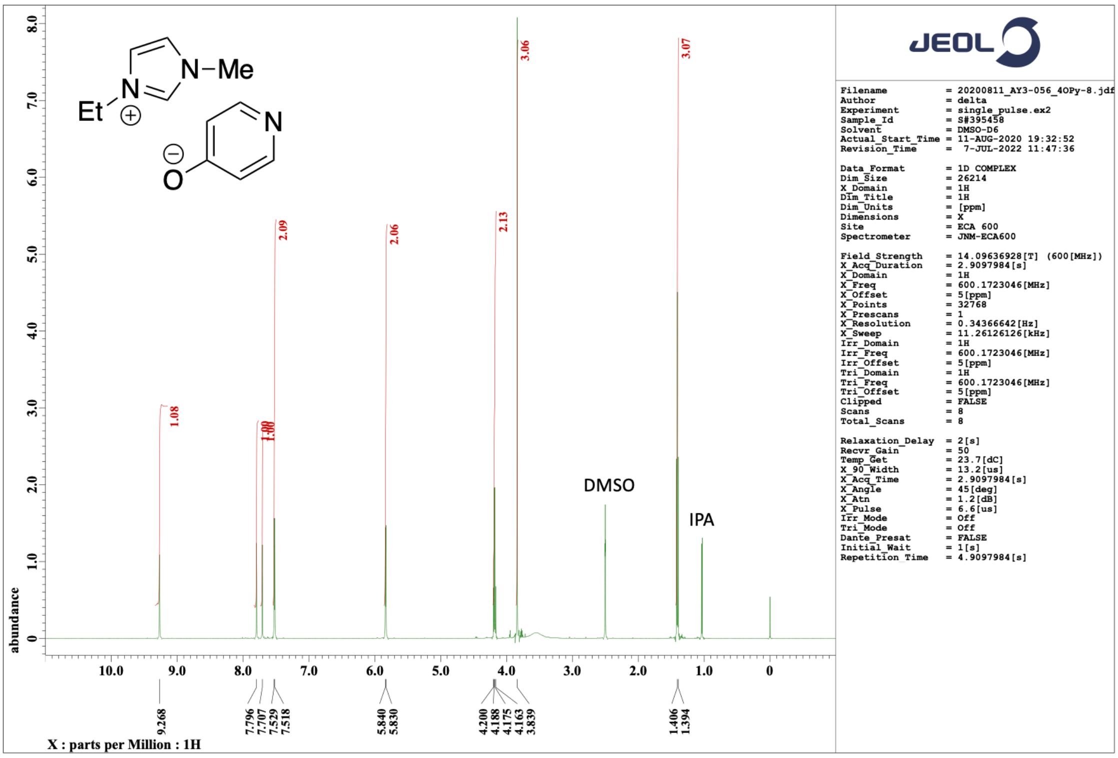

Figure S7. ^{1}H NMR spectrum of 1-ethyl-3-methylimidazolium 4-pyridinolate (Emim4OPy) (**8**) in DMSO-d_6 at rt.

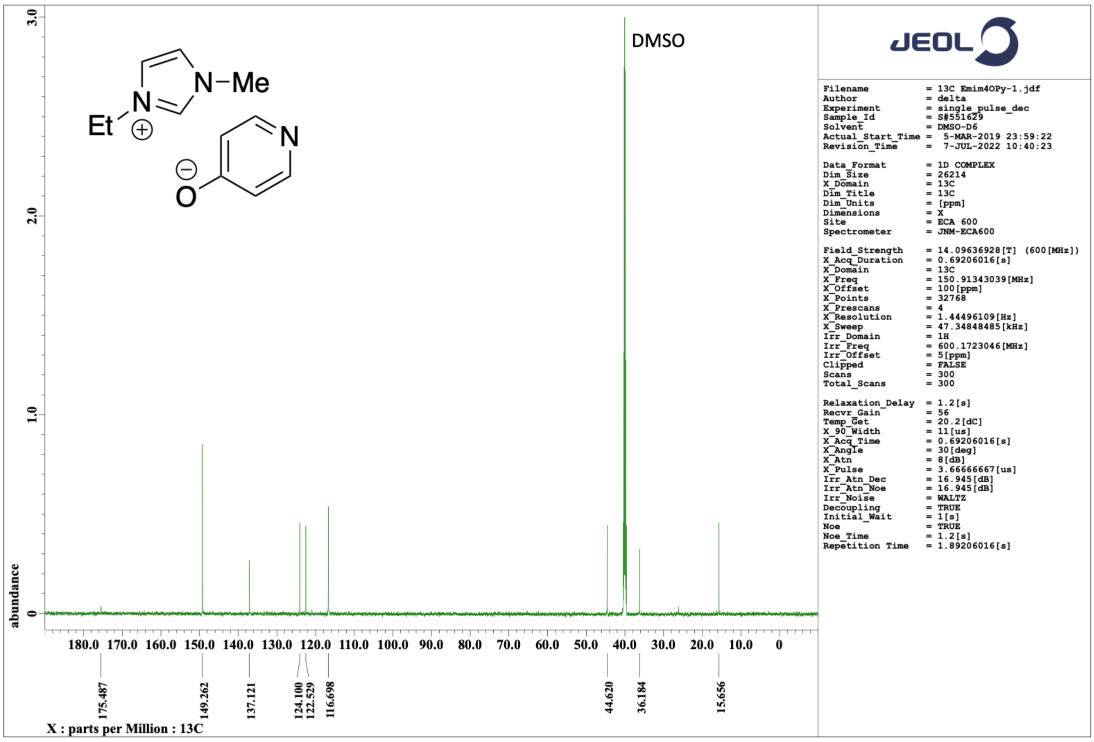

Figure S8. ^{13}C NMR spectrum of 1-ethyl-3-methylimidazolium 4-pyridinolate (Emim4OPy) (**8**) in DMSO-d_6 at rt.

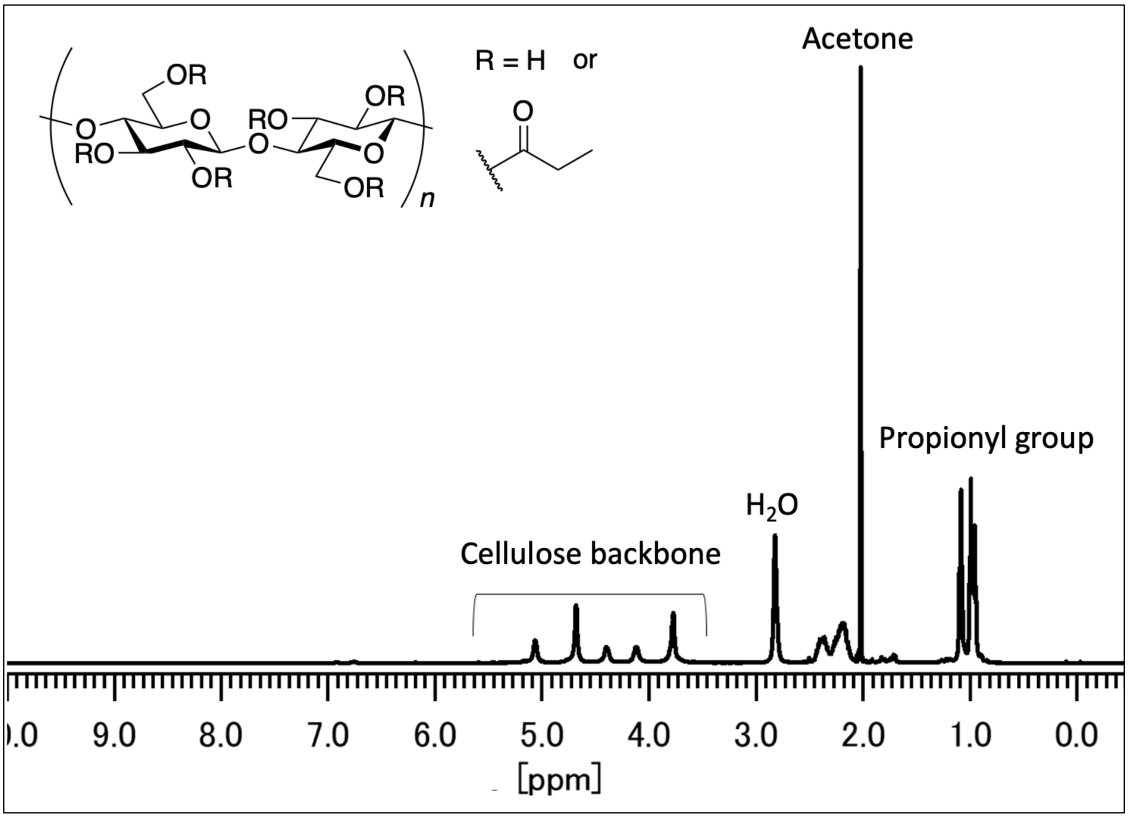

Figure S9. ^{1}H NMR spectrum of cellulose propionate (DS = 2.85) in $CDCl_3$ at rt (Table 3, entry 3).

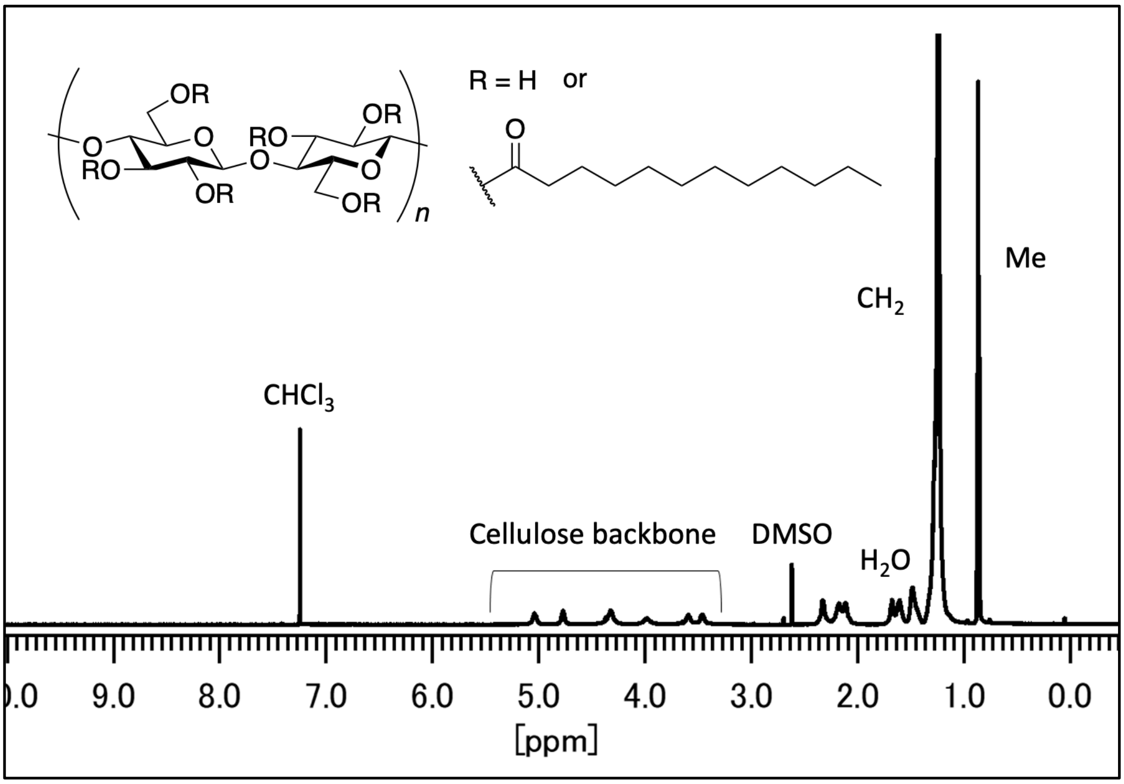

Figure S10. ^{1}H NMR spectrum of cellulose laurate (DS = 2.96) in $CDCl_3$ at rt (Table 3, entry 4).

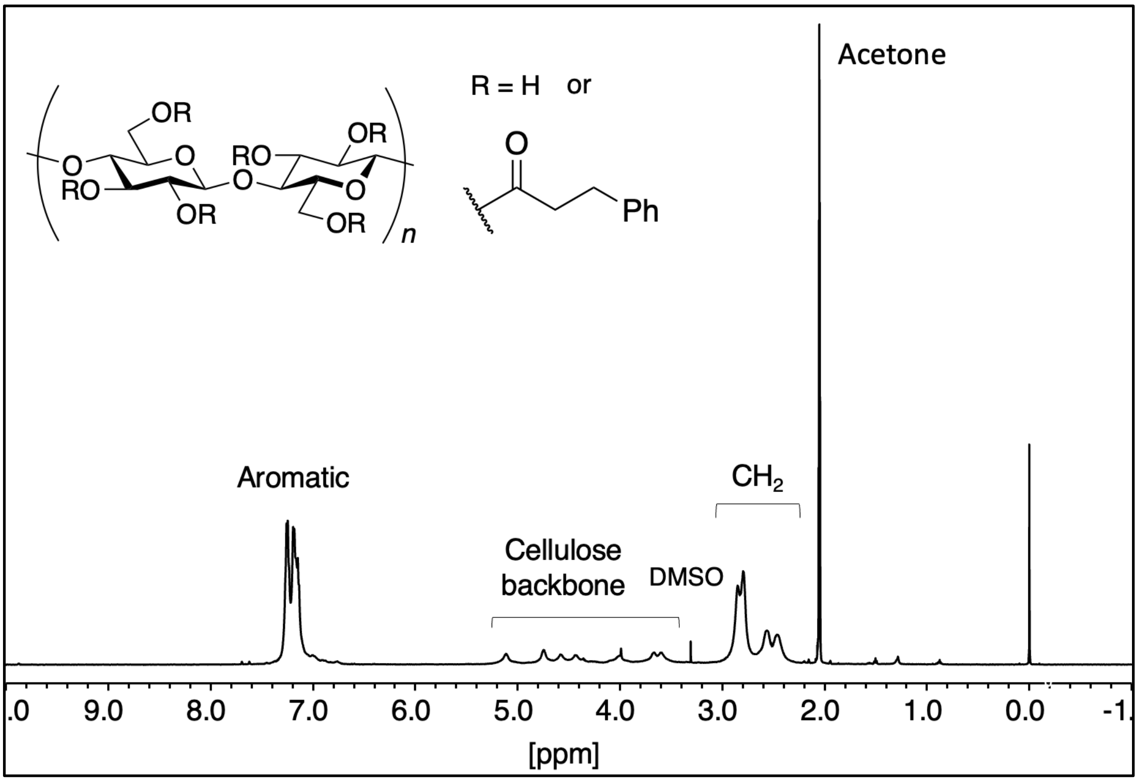

Figure S11. ^{1}H NMR spectrum of cellulose phenylpropionate (DS = 2.78) synthesized from Emim2OPy **6** in acetone-d_6 at rt (Figure 5, blue, 24h).

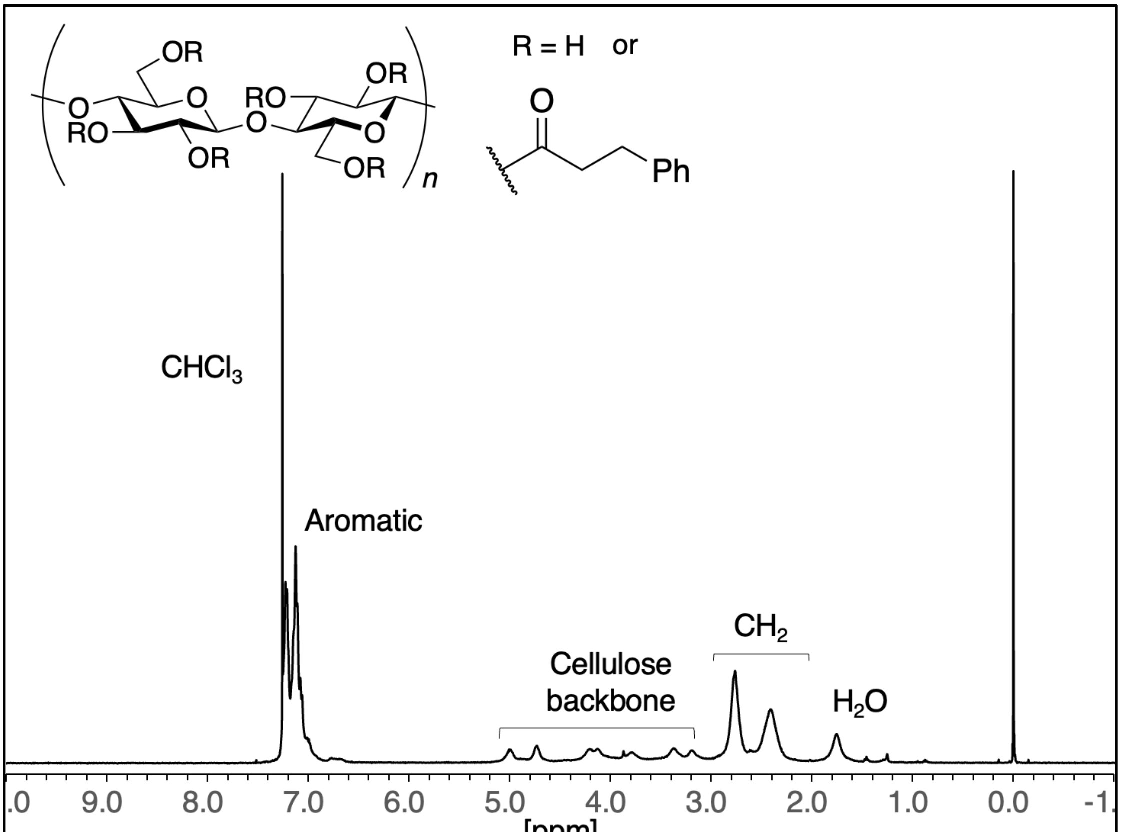

Figure S12. ^{1}H NMR spectrum of cellulose phenylpropionate (DS = 2.78) synthesized from Emim3OPy **7** in $CDCl_3$ at rt (Figure 5, orange, 24h).

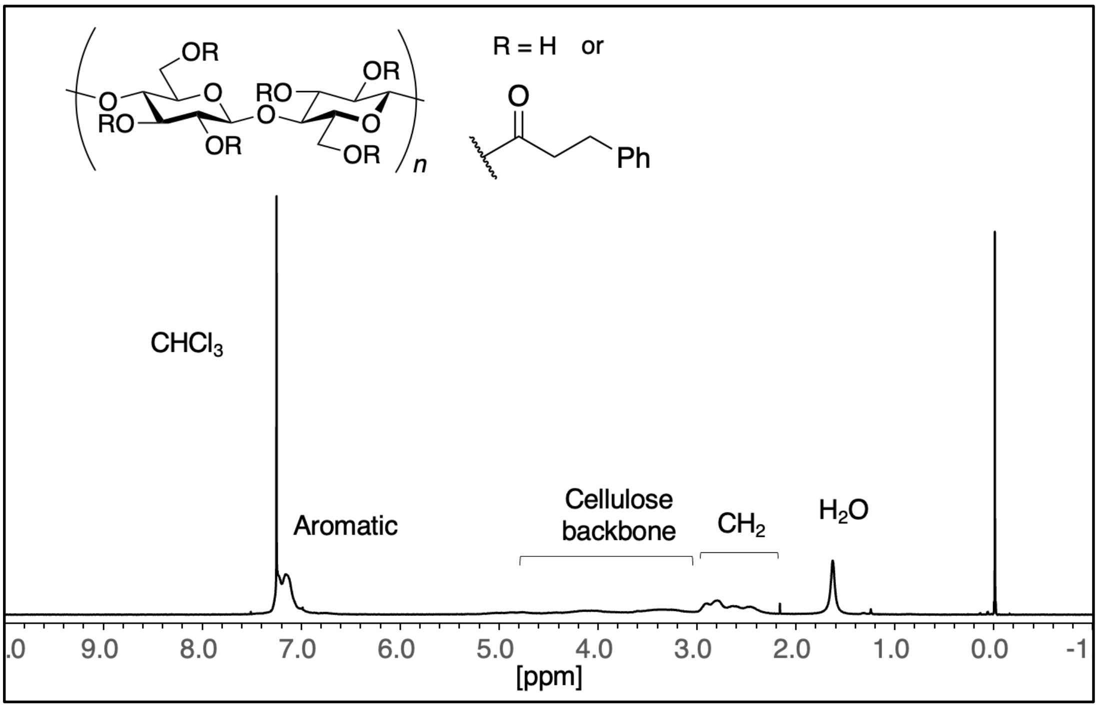

Figure S13. ^{1}H NMR spectrum of cellulose phenylpropionate (DS = 2.60) synthesized from Emim4OPy **8** in $CDCl_3$ at rt (Figure 5, gray, 24h).

Table S1. Solubility test (1.0 mg/mL) of resulting cellulose esters (Table 3) at room temperature.

	Toluene	$CHCl_3$	THF	Acetone	DMF	DMSO	MeOH	H_2O
Entry 1	○	○	○	○	○	○	X	X
Entry 3	X	○	○	○	○	○	X	X
Entry 4	○	○	○	○	○	○	X	X

○: Soluble, X: Insoluble (lower than 1.0 mg/mL)
